# Supplementary material for: Structure of the class C orphan GPCR GPR158 in complex with RGS7-Gβ5
Source: Nat Commun. 2021 Nov 23;12:6805. doi: 10.1038/s41467-021-27147-1 (PMC8611064; doi:10.1038/s41467-021-27147-1)
Supplement: Supplementary file 1 — Supplementary Information [file 41467_2021_27147_MOESM1_ESM.pdf]

SUPPLEMENTARY INFORMATION

Structure of the class C orphan GPCR GPR158 in complex with RGS7-Gβ5

Eunyoung Jeong, Yoojoong Kim, Jihong Jeong and Yunje Cho

Contents

Supplementary Fig. 1. Purification and workflow of cryo-EM processing for apo GPR158.

Supplementary Fig. 2 Analysis of the quality of the cryo-EM map for apo GPR158.

Supplementary Fig. 3. Structural comparison of the PAS domain.

Supplementary Fig. 4. Secondary structure and orthologous conservation of GPR158.

Supplementary Fig. 5. Structural comparison of the CR domain.

Supplementary Fig. 6. Comparison of the GPR158 TM domain with other GPCRs.

Supplementary Fig. 7. Sequence conservation of TM domains from class C GPCRs.

Supplementary Fig. 8. Purification and workflow for cryo-EM processing of the GPR158-RGS7-Gβ5 complex.

Supplementary Fig. 9. Analysis of the quality of the cryo-EM map for GPR158-RGS7-Gβ5 complexes.

Supplementary Fig. 10. Structures of the GPR158-RGS7-Gβ5 complexes.

Supplementary Fig. 11. Importance of the GPR158 TM structure in localizing the RGS7-Gβ5 complex.

Supplementary Fig. 12. Adenylate cyclase activation cell-based assay.

Supplementary Table 1. Cryo-EM data collection, refinement and validation statistics.

Supplementary Table 2. Primer list.

Supplementary References.

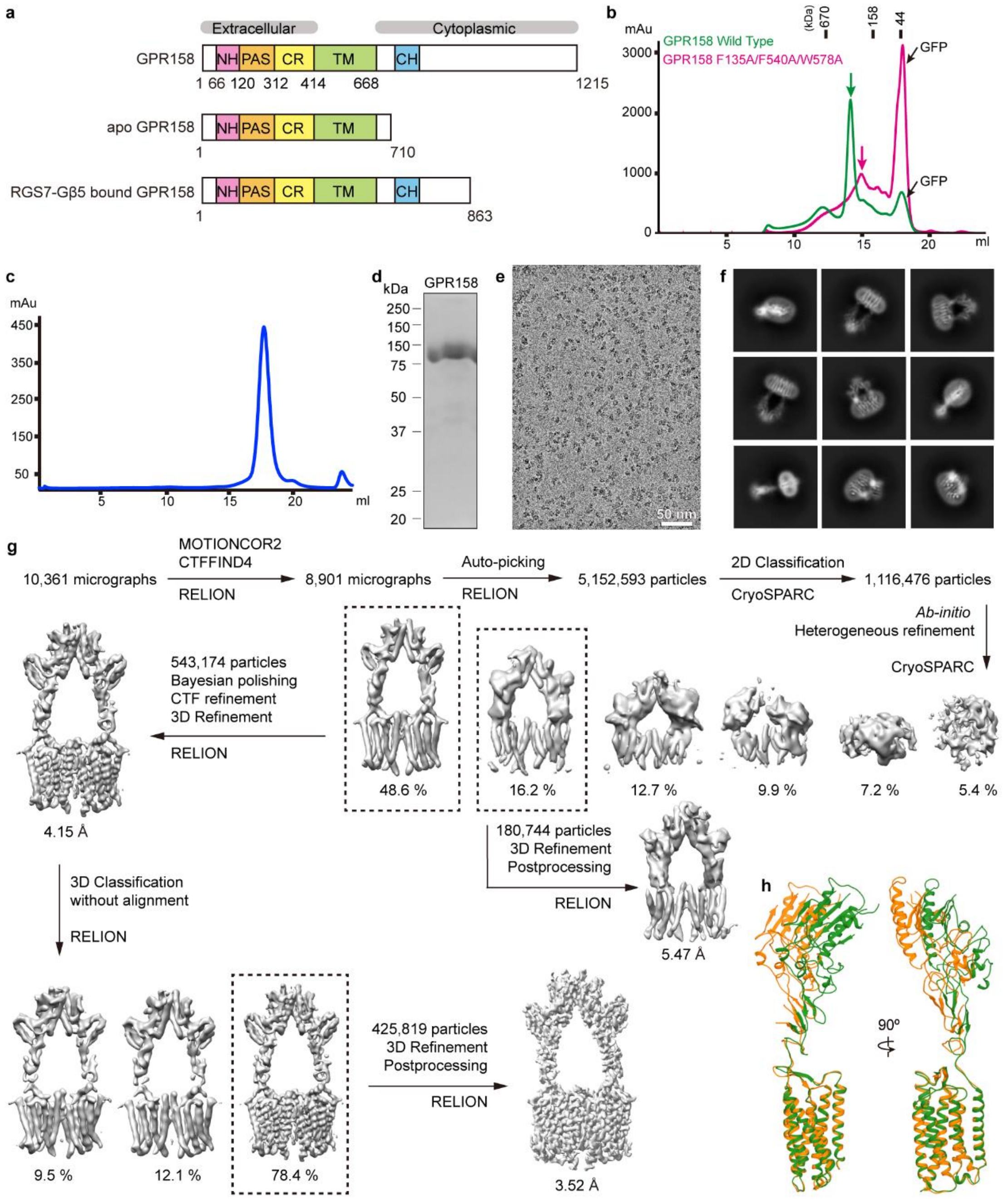

Supplementary Fig. 1 Purification and workflow of cryo-EM processing for apo GPR158. **a** Schematic diagram of subdomain compositions in GPR158 and the constructs used in this study. The N-terminal helices (NH), PAS domain, CR domain, TM domain and Cytoplasmic helices (CH) are colored pink, orange, yellow, green and blue, respectively. **b** Fluorescence size exclusion chromatography profile of wild type and mutant GPR158 (full length). Each

peak corresponding to the GFP-fused GPR158 fraction is indicated by an arrow. **c** Size exclusion chromatography profile of apo GPR158 (residues 1-710). **d** SDS-PAGE analysis of purified apo GPR158 (residues 1-710). **e** Representative image of a raw micrograph from 10,361 movies in vitrified ice. These data were repeated independently three times with similar results (**d, e**). **f** Representative image of 2D class averages from reference-free alignment and classification. **g** Flowchart for single-particle cryo-EM processing. **h** Comparison of the two GPR158 protomers (orange and green) by aligning the TM domains.

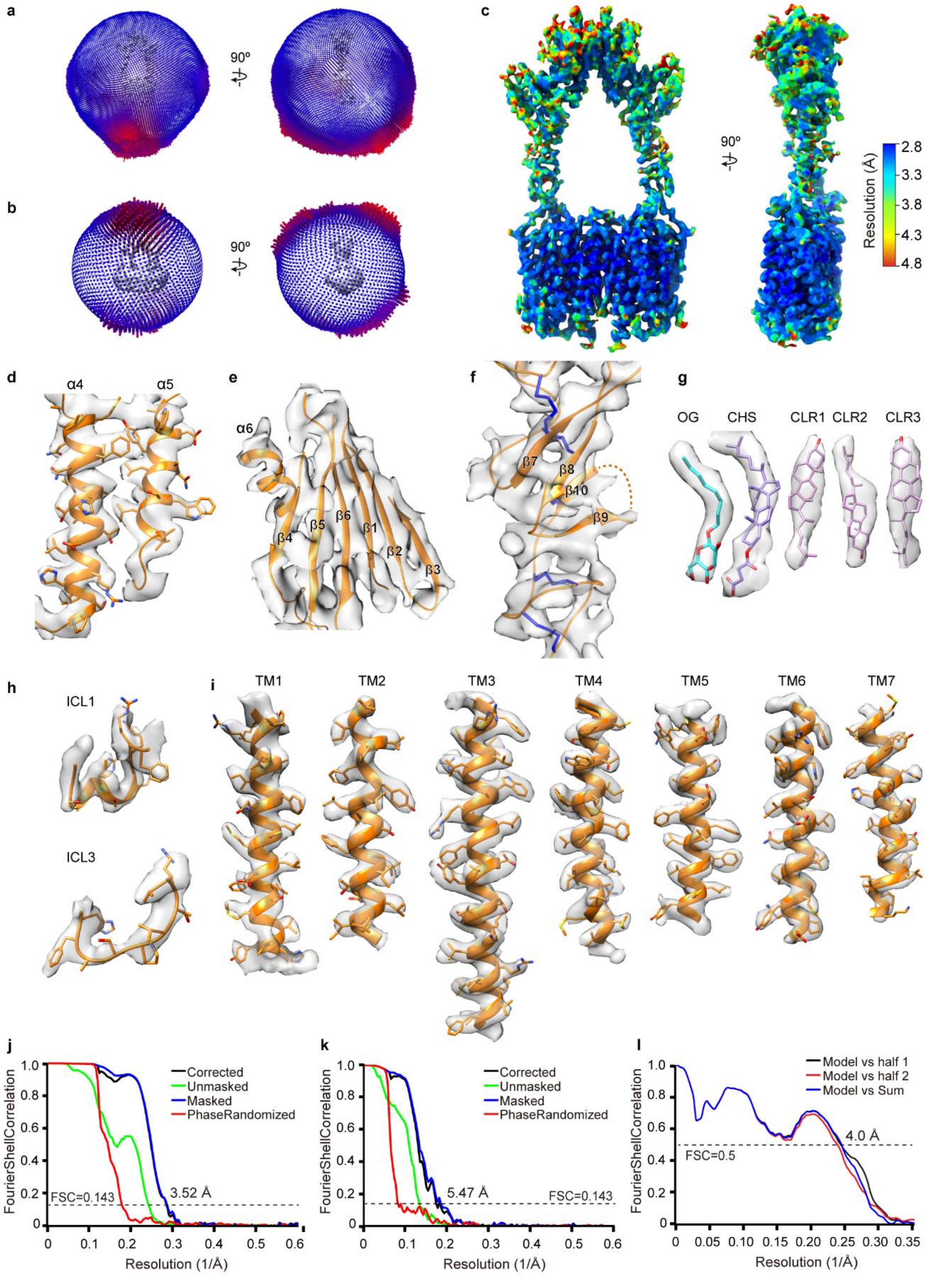

Supplementary Fig. 2 Analysis of the quality of the cryo-EM map for apo GPR158. a, b Angular distribution of the final reconstruction of the maps at 3.52 Å (**a**) and 5.47 Å (**b**). **c**

Local resolution of the map at 3.52 Å. **d-i** Refined model fit of the indicated region in the cryo-EM map at 3.52 Å. **j, k** Gold standard Fourier shell correlation curves for globally refined maps at 3.52 Å (**j**) and 5.47 Å (**k**). **l** Fourier shell correlation curves between the maps at 3.52 Å and the model.

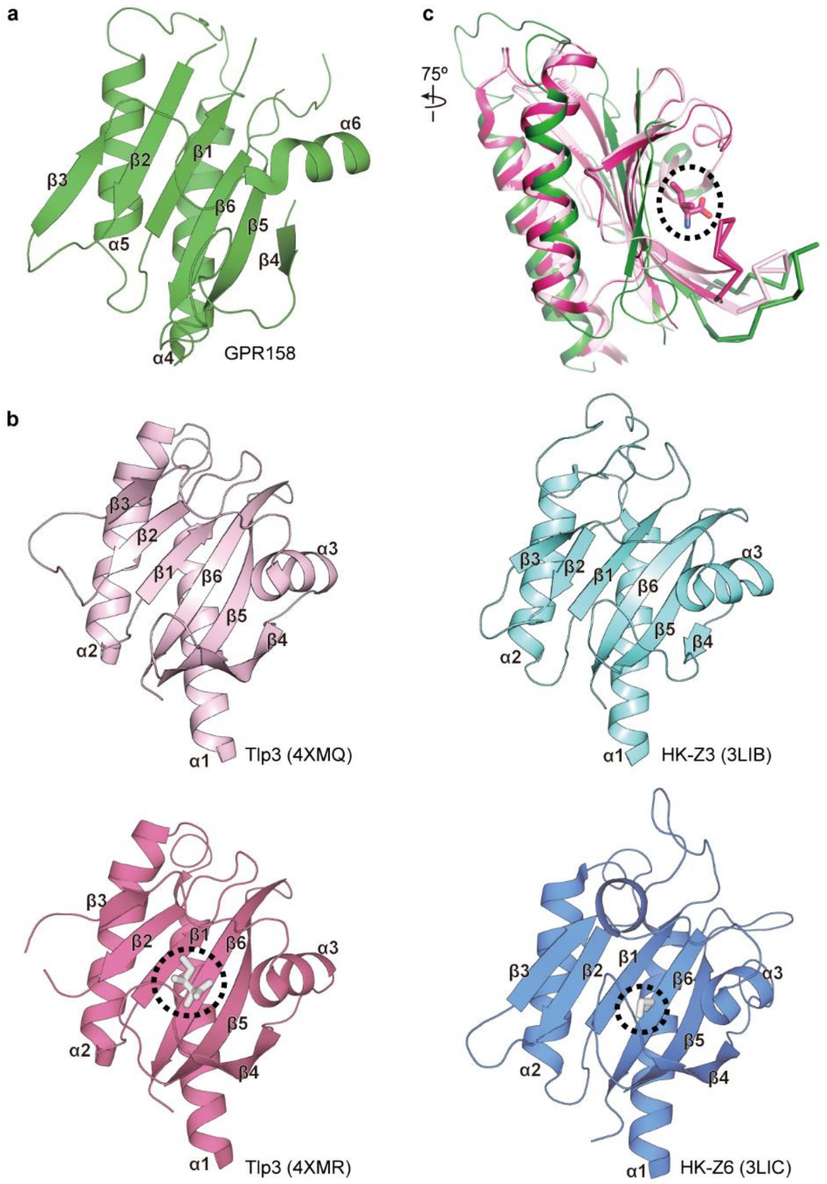

Supplementary Fig. 3 Structural comparison of the PAS domain. a Overall structure of the PAS domain of GPR158 in the view from Fig. 2a. **b** Homologous PAS domain structures from DALI searches: apo (top left) and Ile-bound Tlp3 (bottom left), apo HK-Z3 (top right) and 1,2-ethandiol-bound HK-Z6 (bottom right). Bound ligands are shown as grey sticks. **c** Structural superposition of the GPR158 PAS domain (green) with apo (pink, 4XMQ) and 1,2-ethandiol-bound (magenta, 4XMR) Tlp3. Ligands bound to Tlp3 are shown as sticks and encircled.

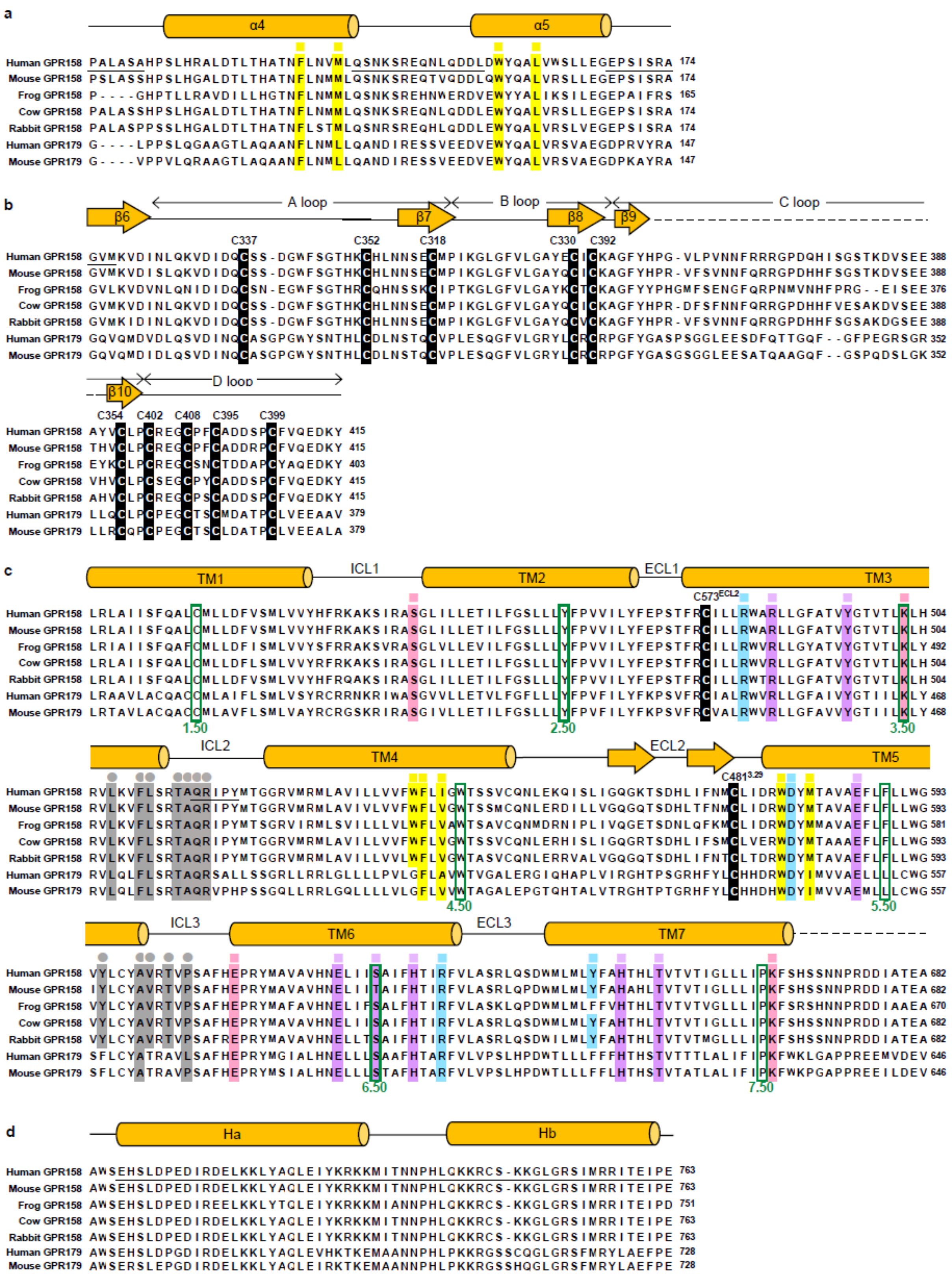

Supplementary Fig. 4 Secondary structure and orthologous conservation of GPR158.

Aligned sequences of GPR158 receptor orthologues; helices α4 and α5 from PAS domain (**a**),

CR domain (**b**), TM domain (**c**) and cytoplasmic coiled-coil (helices Ha and Hb) (**d**). Helices and sheets are indicated as cylinders and arrows, respectively. Segments lacking regular secondary structures and disordered regions are represented as solid lines and dotted lines, respectively. The underlined sequences were built as poly-alanine chains. Cysteines that form disulfide bonds are shaded in black, and paired cysteines are indicated on top. Generic residue numbers based on the Pin numbering scheme[1] in each 7TM helix are colored green. Residues involved in interactions of the dimeric interface, layers I, II, III, and DHEX are colored yellow, blue, purple, pink, and grey, respectively.

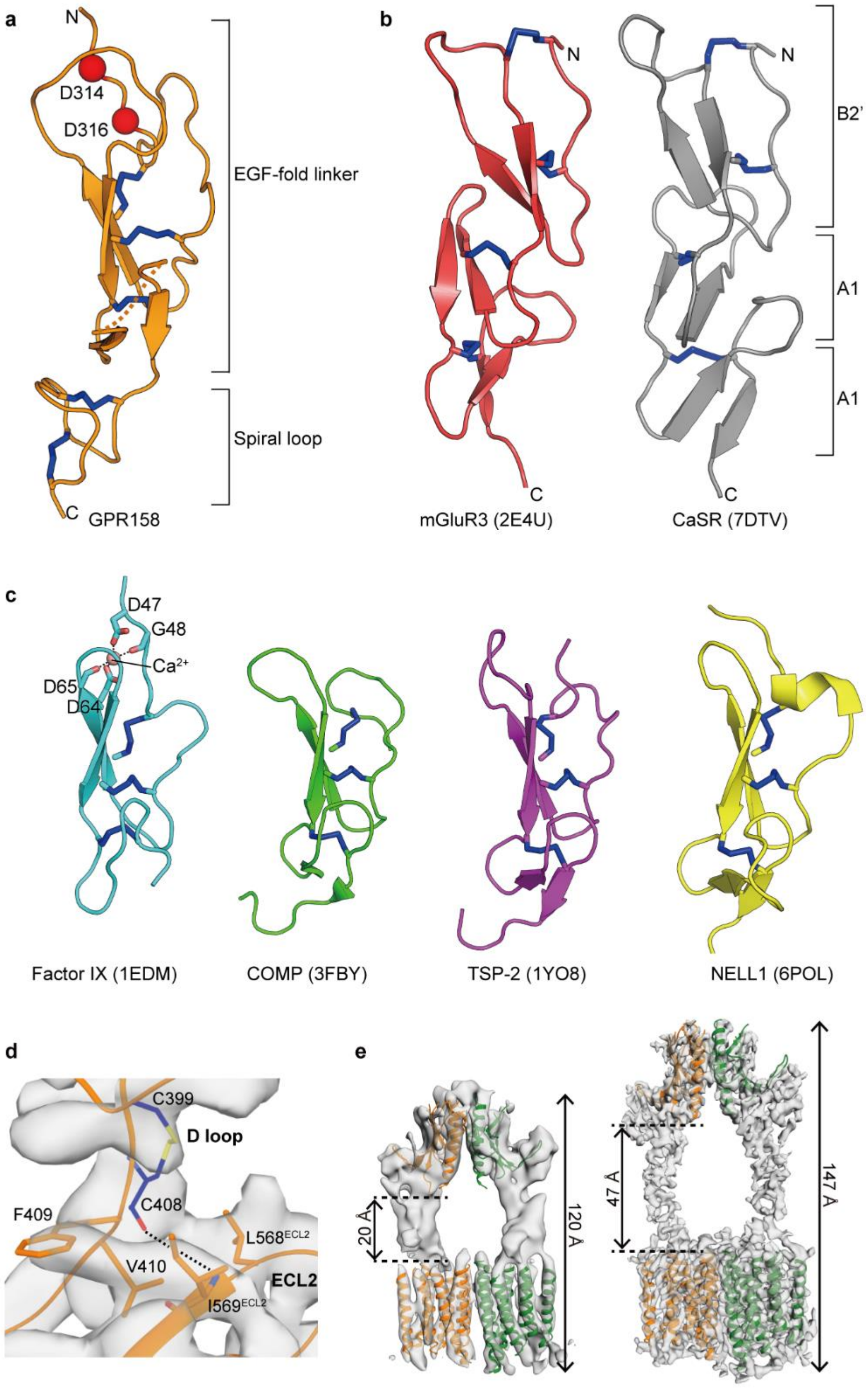

Supplementary Fig. 5 Structural comparison of the CR domain. **a** The CR domain of GPR158 showing the disulfide bonds (blue) and putative Ca^{2+}-binding residues (red spheres). The disordered C loop is shown as a dotted line. **b** The CR domains of mGluR3 (left) and CaSR (right) form the TNF-fold with two A1 modules and a B2' module instead of the EGF-like fold. **c** DALI search structures similar to the GPR158 EGF-like linker (ordered left to right): Factor IX, cartilage oligomeric matrix protein (COMP), thrombospondin-2 (TSP-2) and neural

epidermal growth factor-like-like 1 (NELL1). Conserved disulfide bonds are shown as blue sticks, and the Ca^{2+} ion in Factor IX is shown as a sphere with coordinating residues in stick representation. **d** The interaction between D loop and ECL2. Hydrophobic interactions are shown as sticks, and the hydrogen bond is represented as a dotted line. The disulfide between Cys399 and Cys408 is displayed as blue sticks. **e** GPR158 model in which the CR domain is compressed by 27 Å. The PAS and TM domains are fitted into the 5.47 Å (left) and 3.52 Å (right) maps.

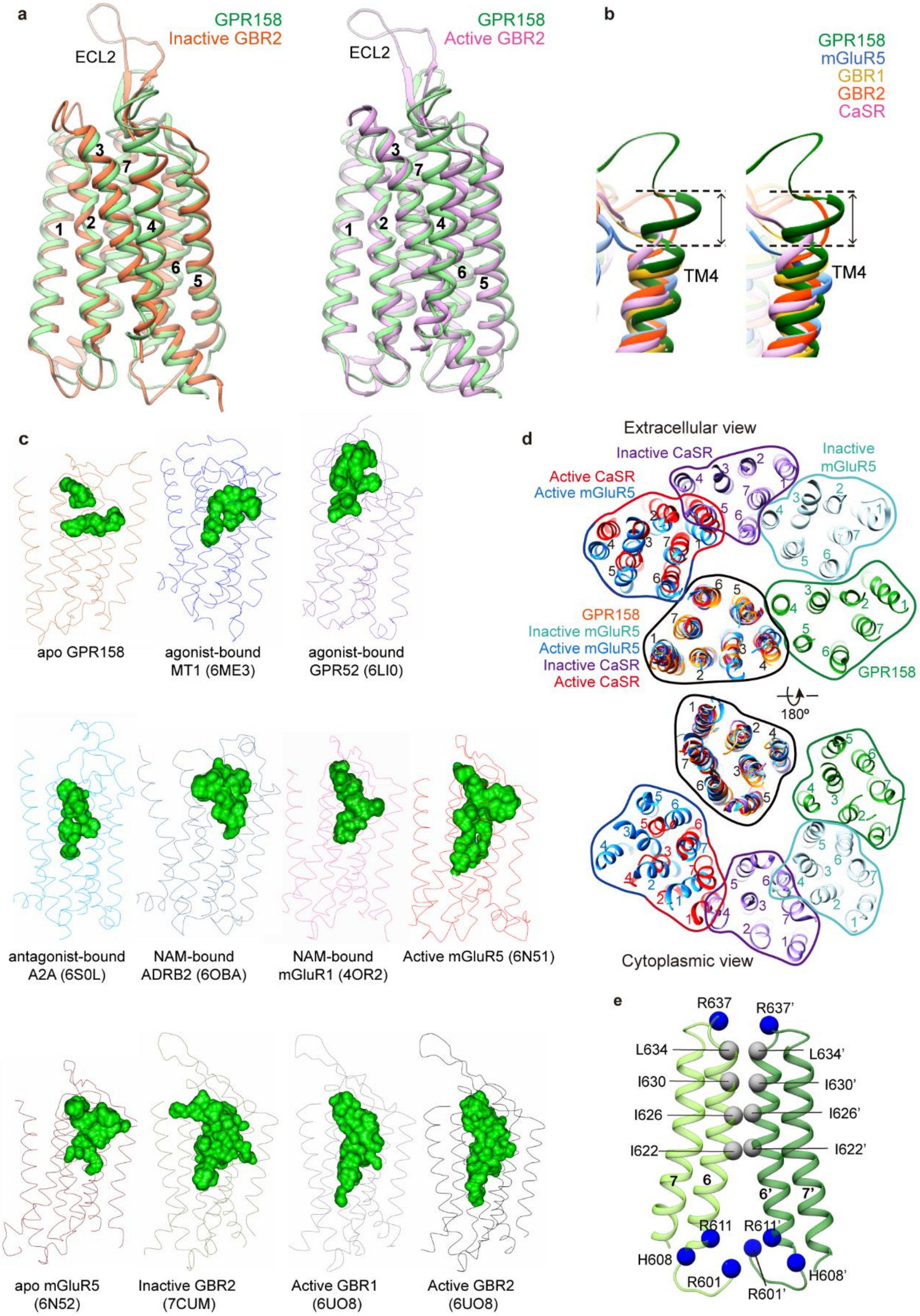

Supplementary Fig. 6 Comparison of the GPR158 TM domain with other GPCRs. a Superposition of the GPR158 TM domain onto the inactive (left) and active (right) GBR2 structures. **b** Alignment of the five TM domains of class C GPCRs in inactive (left) and active

(right) states. The TM4 helix of GPR158 is one and a half turns longer (arrows) at the extracellular end than at other TM domains. **c** Computation of the cavity within the TM domains of the ligand-free class A and C GPCRs performed using Caver 2.0[2]. Each cavity is colored light green. **d** Comparison of the dimeric interface of GPR158 TM domains with those of mGluR5 and CaSR in inactive and active states. The TM domains of mGluR5 and CaSR in inactive and active states are superposed onto one protomer of GPR158 (black line), and are shown in extracellular (top) and cytoplasmic (bottom) views. **e** Alignment of each TM domain of GPR158 with active GBR1 and GBR2 to reconstitute the TM6/7-TM6'/7' interface. Hydrophobic and positively charged residues involved in interactions at the interface are shown as grey and blue spheres, respectively.

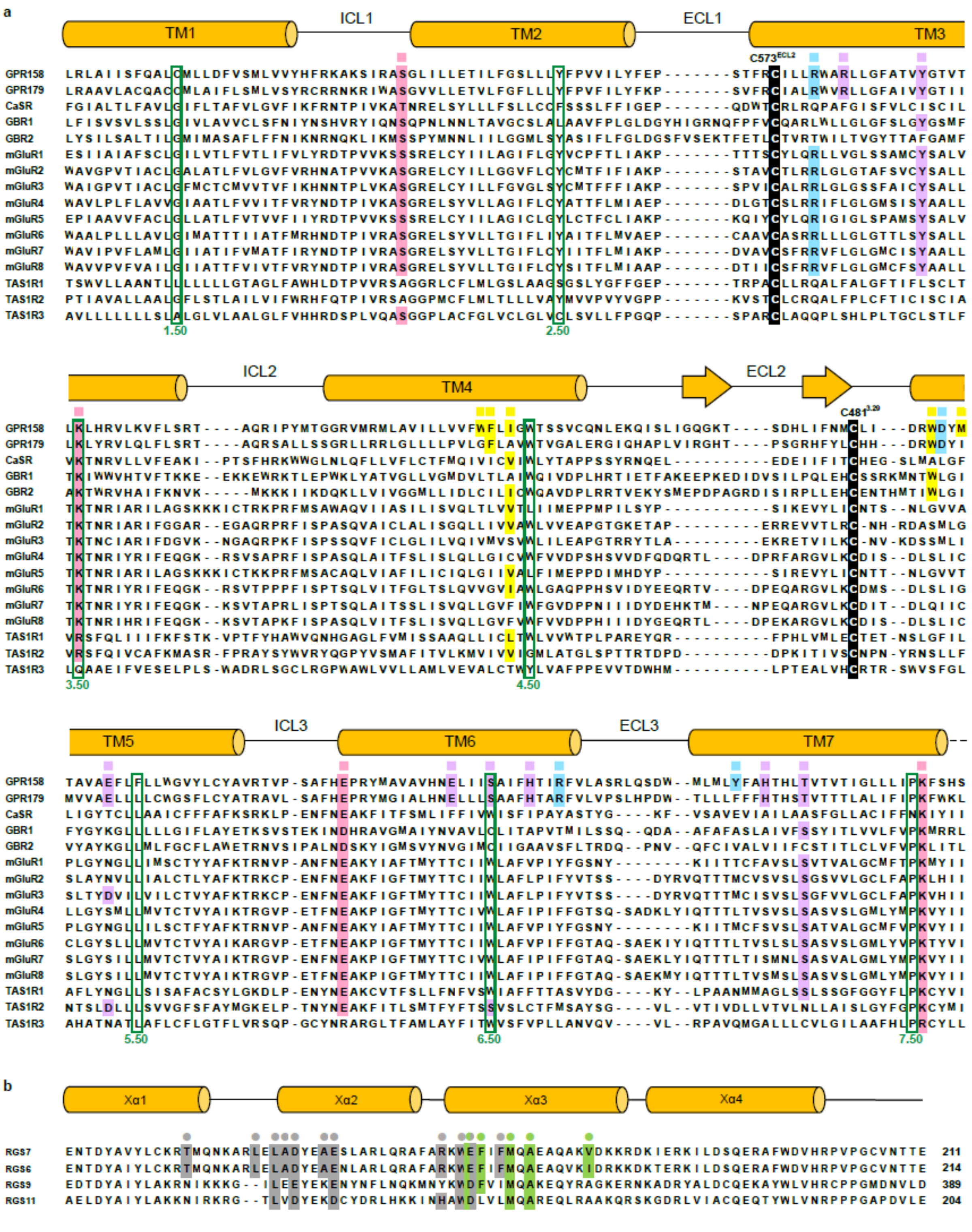

Supplementary Fig. 7 Sequence conservation of TM domains from class C GPCRs. a Aligned sequences of the TM domains from class C GPCRs displayed in the same way as Supplementary Fig. 3. **b** Alignment of sequences of the DHEX domains of R7 family proteins. Helices are indicated as cylinders. Residues in close proximity to GPR158 TM domains and

cytoplasmic helices are colored grey and green, respectively.

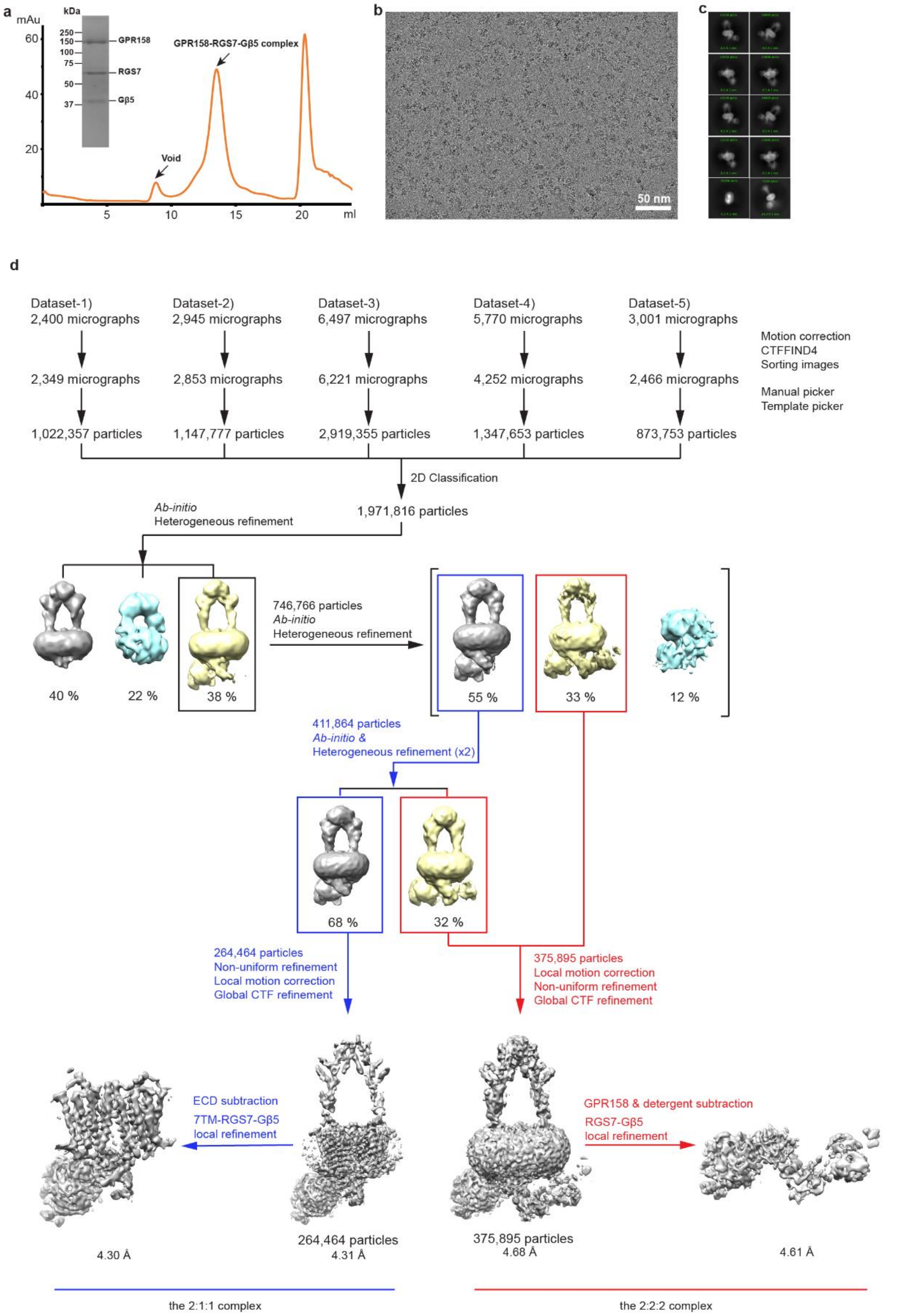

Supplementary Fig. 8 Purification and workflow for cryo-EM processing of the GPR158-RGS7-Gβ5 complex. a Size exclusion chromatography profile and SDS-PAGE analysis of the GPR158-RGS7-Gβ5 complex. **b** Representative cryo-EM micrograph from 20,613 movies of

the GPR158-RGS7-Gβ5 complex. These data were repeated independently three times with similar results (**a, b**). **c** Representative 2D class averages of the GPR158-RGS7-Gβ5 complex. **d** Flowchart for single particle cryo-EM processing.

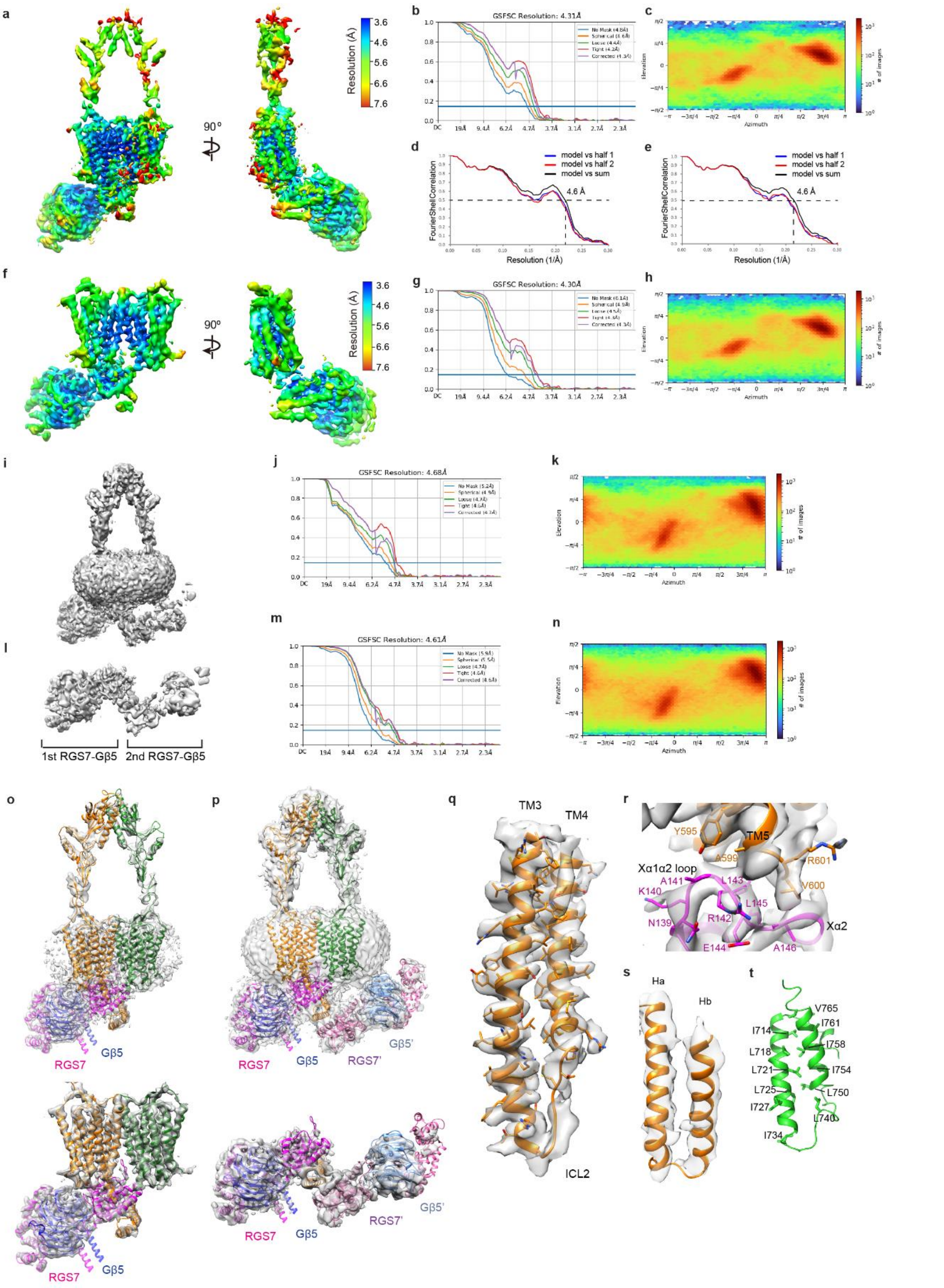

Supplementary Fig. 9 Analysis of the quality of the cryo-EM map for GPR158-RGS7-Gβ5 complexes. a−c, Local resolution **(a)**, Gold-standard Fourier shell correlation curves (No mask, blue; Spherical, orange; Loose, green; Tight, red; Corrected, purple) **(b)**, and angular

distribution of particles for the overall refined 2:1:1 complex of GPR158-RGS7-Gβ5 **(c)**. **d, e** Fourier shell correlation curves between the maps for overall refined **(d)** and locally refined **(e)** 2:1:1 complex and the corresponding models. **f–h,** Local resolution **(f)**, FSC curves (No mask, blue; Spherical, orange; Loose, green; Tight, red; Corrected, purple) **(g)**, and angular distribution of particles for the locally refined 2:1:1 complex **(h)**. **i–k,** An overall refinement map **(i)**, FSC curves (No mask, blue line; Spherical, orange; Loose, green; Tight, red; Corrected, purple) **(j)**, and angular distribution of particles for overall refined 2:2:2 complex of GPR158-RGS7-Gβ5 **(k)**. **l-n,** A locally refined map **(l)**, FSC curves (No mask, blue; Spherical, orange; Loose, green; Tight, red; Corrected, purple) **(m)**, and angular distribution of particles for locally refined two RGS7-Gβ5 heterodimers and the cytoplasmic helices **(n)**. **o, p** Models of the overall structures for the 2:1:1 complex (**o**) and the 2:2:2 complex **(p)** in overall refined maps (top) and locally refined maps (bottom). **q–s,** Cryo-EM map at 4.31 Å and a model of the ICL2, TM3 and TM4 **(q)**, the Xα1α2 loop **(r)**, and cytoplasmic coiled-coil **(s)**. **t,** A 3D model of the cytoplasmic coiled-coil region (residues N701 to S769) constructed using the I-TASSER server[3].

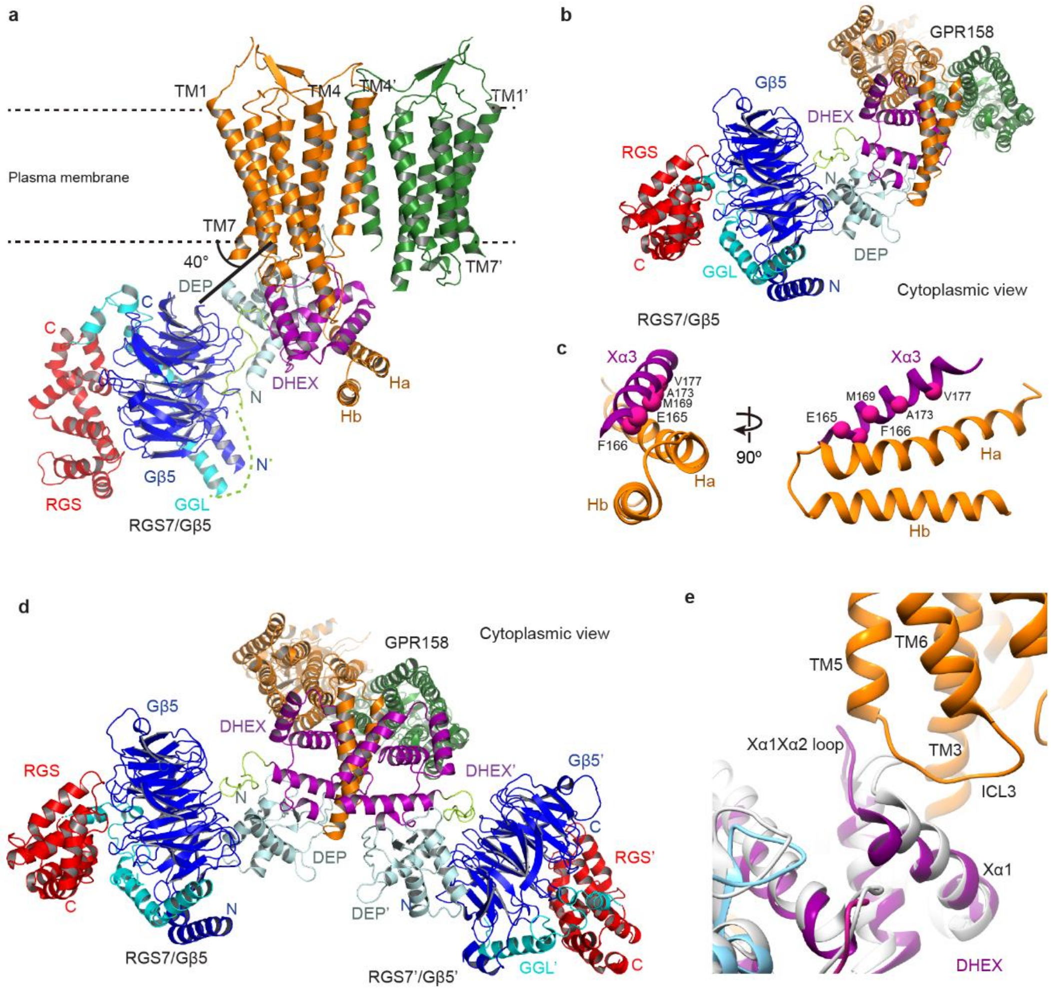

Supplementary Fig. 10 Structures of the GPR158-RGS7-Gβ5 complexes. a, b Orthogonal views of representative cryo-EM maps and models for the 2GPR158-2RGS7-2Gβ5 complex. The models are colored as follow; each 7TM domain of GPR158, orange and green, respectively; DEP domain, light cyan; DHEX, purple; GGL-RGS linker, light green; GGL, cyan; RGS domain, red; Gβ5, blue. **c** Interactions between Xα3 and the Ha helix in two views. Indicated residues (spheres) of the Xα3 helix of the DHEX domain make contacts with the Ha helix. **d** Representative model for the 2GPR158-2RGS7-2Gβ5 complex in the cytoplasmic view of Fig.5a. Each domain is colored as in Fig. 5a. **e** Structural comparison of the RGS7 (purple) bound to GPR158 (orange) with the crystal structure of RGS9 (PDB 2PBI, Grey) by aligning the DHEX domain. The Xα1 helix and Xα1Xα2 loop of DHEX cause steric collision with ICL3 and TM3, respectively. The DEP domain of RGS7 is colored light cyan.

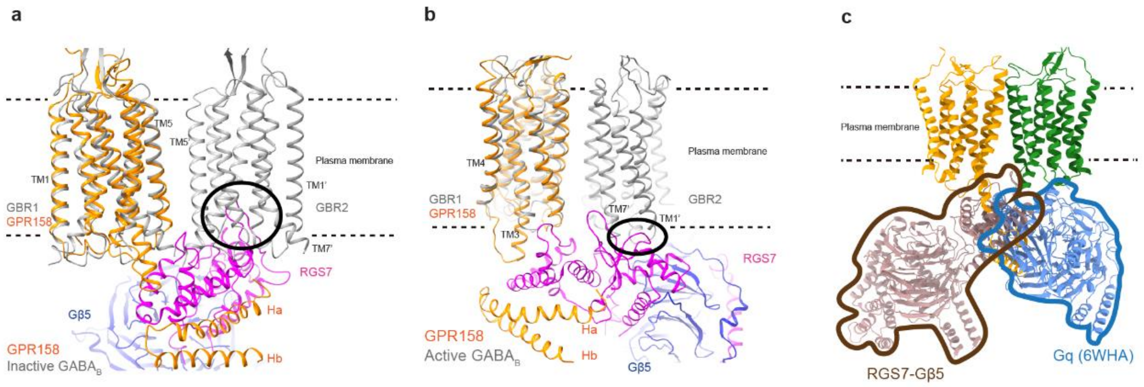

Supplementary Fig. 11 Importance of the GPR158 TM structure in localizing the RGS7-Gβ5 complex. a, b The GPR158 TM domain (orange) in complex with RGS7 (magenta)-Gβ5 (blue) is superimposed with inactive (**a**) or active (**b**) GBR1 TM domain (grey). Each GBR2 TM domain (grey) collides to RGS7, which is indicated by a circle. **c** A model for the 2GPR158-RGS7-Gβ5 complex directly coupled to the Gq protein. The Gq protein (PDB 6WHA, blue) was modelled by aligning the TM domain of 5-HT2A to the TM domain of GPR158 (green). A modelled Gq protein collides to the RGS7 complex. The TM domain of another GPR158 protomer is colored orange, and RGS7-Gβ5 is colored brown.

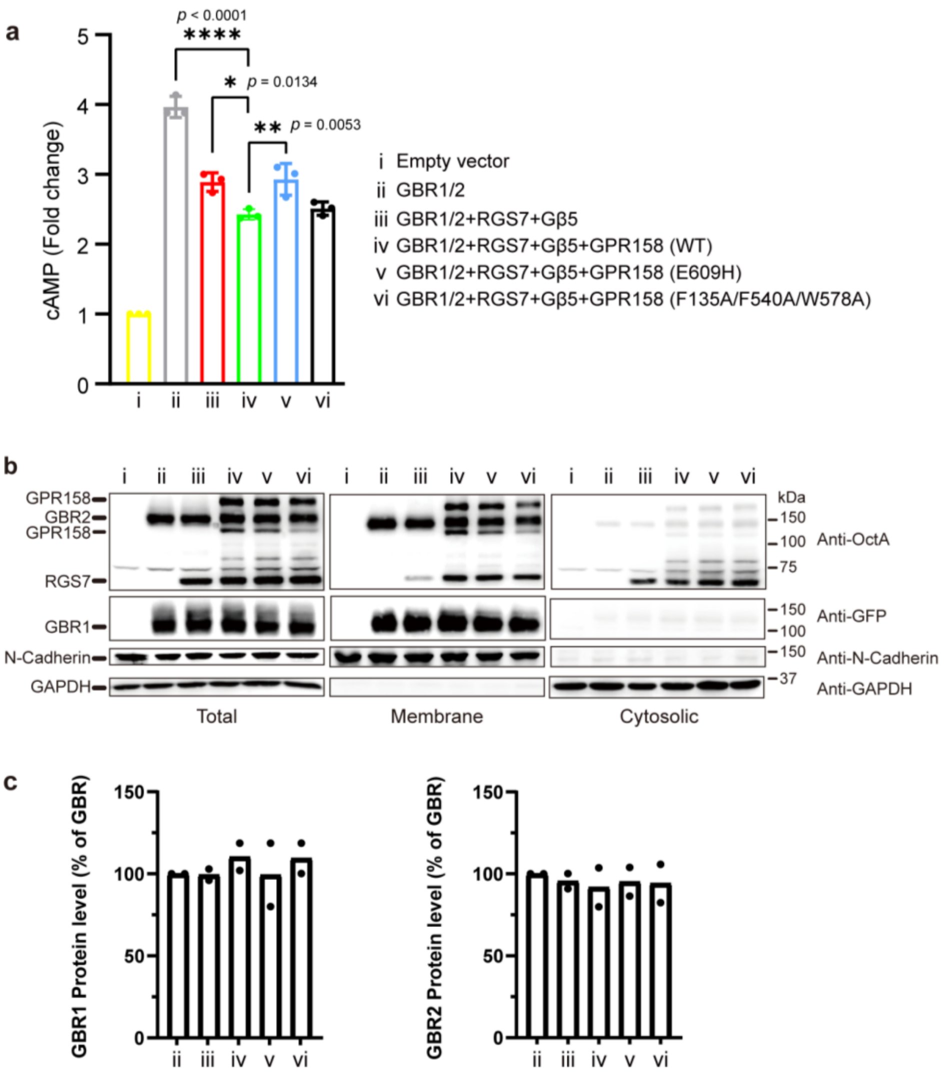

Supplementary Fig. 12 Adenylate cyclase activation cell-based assay. a cAMP levels at 4.5 min after CGP54626 application in each condition. The figure is related to Fig. 5e. Data are mean values with standard deviations from three independent experiments and were analyzed one-way ANOVA and Tukey's post-hoc test ($n = 3$; $F_{5, 12} = 157.2$; *$p < 0.05$, **$p < 0.01$, ***$p < 0.001$, ****$p < 0.0001$). **b** Subcellular fractionation was performed to examine the expression level and localization of GBR1-GFP, GBR2-Flag, GPR158-Flag and RGS7-Flag. Western blot anlysis of total (left), membrane (middle) and cytosolic (right) fractions of four proteins. GAPDH and N-cadherin represent markers for cytosolic and membrane fractions, respectively. These data were repeated from two independent experiments. **c** Quantification of GBR1 (left)

and GBR2 (right) band densities from the membrane fraction of Western blot analysis (**b**). Data are mean values with standard deviations from two independent experiments.

Supplementary Table 1 | Cryo-EM data collection, refinement and validation statistics.

	apo GPR158 (EMDB-31351) (PDB 7EWL)	[a]2GPR158-RGS7-Gβ5 ([b]EMDB-31360, [c]EMDB-31365) (PDB 7EWP)	[a]2GPR158-2RGS7-2Gβ5 ([b]EMDB-31363, [c]EMDB-31366) (PDB 7EWR)
Magnification	100,000	79,000	
Voltage (kV)	200	200	
Electron exposure (e–/Å^2)	50	50	
Defocus range (μm)	−0.6 to −1.2	−1.0 to −2.0	
Pixel size (Å)	0.83	1.06	
Symmetry imposed	C1	C1	C1
Initial particle images (no.)	5,152,593	5,089,489	5,089,489
Final particle images (no.)	425,819	264,464	375,895
Map resolution (Å)	3.5	4.3	4.7
FSC threshold	0.143	0.143	0.143
Initial model used (PDB code)	-	6N9G	
Model resolution (Å)	4.0	4.6	
FSC threshold	0.5	0.5	
Map sharpening *B* factor (Å^2)	-142.7	-163.0	
Model composition			
Non-hydrogen atoms	7,035	13,383	
Protein residues	994	1,816	
B factors (Å^2)			
Protein	34.76	24.19	
R.m.s. deviations			
Bond lengths (Å)	0.008	0.012	
Bond angles (°)	1.019	1.666	
Validation			
MolProbity score	1.74	2.13	
Clashscore	3.54	9.65	
Poor rotamers (%)	0.65	0.87	
Ramachandran plot			
Favored (%)	88.33	87.11	
Allowed (%)	11.67	12.89	
Disallowed (%)	0	0	

[a] Same data set was used for GPR158-RGS7-Gβ5 complexes

[b] Overall refined map data were used for refinement.

[c] Locally refined map data

Supplementary Table 2 | Primer list.

Name	Sequence
GPR158 F	ACGCGTCGACGCCACCATGGGCGCTATGGCATACC
GPR158 710 R	CTGGCGGCCGCGGTCGAGACTGTGCTCGCTC
GPR158 863 R	CTGGCGGCCGCGTTCTTTCAATTTCTGGGTCAGTTTCT
GPR158 full length R	CTGGCGGCCGCGTACTTTAAAGGAGTCCCAGATCTC
GPR158 F135A F	GACCCATGCAACTAACGCTCTTAATGTTATGCTTC
GPR158 F135A R	GAAGCATAACATTAAGAGCGTTAGTTGCATGGGTC
GPR158 F540A F	TATTCTGGGCCCTGATCGGCTGGACCAG
GPR158 F540A R	CCGATCAGGGCCCAGAATACTACAAGCAGTATGAC
GPR158 W578A F	TAGACAGGGCCGATTACATGACAGCCGTGGC
GPR158 W578A R	CATGTAATCGGCCCTGTCTATCAAGCACATGTTGA
GPR158 E609H F	GTTCCACCACCCCCGATACATGGCCGT
GPR158 E609H R	TCGGGGGTGGTGGAACGCGGAAGGGA
RGS7 F	ACGCGTCGACGCCACCATGGCCCAGGGGAATAATTATG
RGS7 R	CTGGCGGCCGCGTAACAGGTTAGTGCTGGCCCT
Gβ5 F	ACGCGTCGACGCCACCATGGCAACCGAGGGGCT
Gβ5 R	CTGGCGGCCGCTTAGGCCCAGACTCTGAGG
TCM GBR1 F	CCGGAATTCGGCCGCCATGAGACTGCTGACCGCCCTGTTCGCC TACTTCATCGTGGCCCTGATCCTGGCCTTCAGCGTG
GBR1 F	TCCTGGCCTTCAGCGTGTCCGCCAAGAGCATGTCCGAGAGACG AGCTGTTTA
GBR1 R	ATAGTTTAGCGGCCGCTTTTCTCCAGTTCCCTATTCTCCTTT
GBR2 F	CCGGAATTCGGCCGCCATGGCTTCCCCCCGC
GBR2 R	ATAGTTTAGCGGCCGCTCAGGCCGGACACCATCA

Supplementary References

1 Pin, J. P., Galvez, T. & Prezeau, L. Evolution, structure, and activation mechanism of family 3/C G-protein-coupled receptors. *Pharmacol Ther* **98**, 325-354 (2003).

2 Jurcik, A. *et al.* CAVER Analyst 2.0: analysis and visualization of channels and tunnels in protein structures and molecular dynamics trajectories. *Bioinformatics* **34**, 3586-3588 (2018).

3 Zhang, Y. I-TASSER server for protein 3D structure prediction. *BMC Bioinformatics* **9**, 40 (2008).
