# Supplementary material for: Structure of the class C orphan GPCR GPR158 in complex with RGS7-Gβ5
Source: Nat Commun. 2021 Nov 23;12:6805. doi: 10.1038/s41467-021-27147-1 (PMC8611064; doi:10.1038/s41467-021-27147-1)
Supplement: Supplementary file 3 — Description of Additional Supplementary Files [file 41467_2021_27147_MOESM3_ESM.pdf]

Description of Additional Supplementary Files

Supplementary Movie 1. Local 3D variability analysis of the 2GPR158-2RGS7-2Gβ5 complex. The 3D variability analysis in cryoSPARC v3.1 reveals dynamic movement of the RGS7-Gβ5 heterodimers, in which the C-terminal region of the second RGS7 complex is directed toward the detergent micelle
