# Supplementary material for: Structure of the class C orphan GPCR GPR158 in complex with RGS7-Gβ5
Source: Nat Commun. 2021 Nov 23;12:6805. doi: 10.1038/s41467-021-27147-1 (PMC8611064; doi:10.1038/s41467-021-27147-1)
Supplement: Supplementary file 5 — Reporting Summary [file 41467_2021_27147_MOESM5_ESM.pdf]

nature portfolio

Corresponding author(s): Yunje Cho

Last updated by author(s): Oct 31, 2021

Reporting Summary

Nature Portfolio wishes to improve the reproducibility of the work that we publish. This form provides structure for consistency and transparency in reporting. For further information on Nature Portfolio policies, see our Editorial Policies and the Editorial Policy Checklist.

Statistics

For all statistical analyses, confirm that the following items are present in the figure legend, table legend, main text, or Methods section.

n/a	Confirmed	
☐	☒	The exact sample size (*n*) for each experimental group/condition, given as a discrete number and unit of measurement
☐	☒	A statement on whether measurements were taken from distinct samples or whether the same sample was measured repeatedly
☐	☒	The statistical test(s) used AND whether they are one- or two-sided<br>*Only common tests should be described solely by name; describe more complex techniques in the Methods section.*
☒	☐	A description of all covariates tested
☒	☐	A description of any assumptions or corrections, such as tests of normality and adjustment for multiple comparisons
☐	☒	A full description of the statistical parameters including central tendency (e.g. means) or other basic estimates (e.g. regression coefficient) AND variation (e.g. standard deviation) or associated estimates of uncertainty (e.g. confidence intervals)
☐	☒	For null hypothesis testing, the test statistic (e.g. *F*, *t*, *r*) with confidence intervals, effect sizes, degrees of freedom and *P* value noted<br>*Give P values as exact values whenever suitable.*
☒	☐	For Bayesian analysis, information on the choice of priors and Markov chain Monte Carlo settings
☒	☐	For hierarchical and complex designs, identification of the appropriate level for tests and full reporting of outcomes
☒	☐	Estimates of effect sizes (e.g. Cohen's *d*, Pearson's *r*), indicating how they were calculated

Our web collection on statistics for biologists contains articles on many of the points above.

Software and code

Policy information about availability of computer code

Data collection	The commercial software EPU v.2.8.0 (Thermo Fisher) was used for cryoEM data collection.
Data analysis	The following software was used in this study: MotionCor2, CTFFIND v4, RELION v. 3.0, CryoSparc v. 2.15 and v. 3.1, Coot v. 0.8.9.1, UCSF ChimeraX v. 0.7.0, UCSF Chimera v. 1.14 and 1.15, PyMOL v. 2.3.0, Phenix v 1.15.2, GraphPad Prism 8.4.3, PSIPRED v4.0, XtalPred RF, CAVER Analyst 2.0, I-TASSER server (https://zhanglab.dcmb.med.umich.edu/I-TASSER/), Dali server (http://ekhidna2.biocenter.helsinki.fi/dali/).

For manuscripts utilizing custom algorithms or software that are central to the research but not yet described in published literature, software must be made available to editors and reviewers. We strongly encourage code deposition in a community repository (e.g. GitHub). See the Nature Portfolio guidelines for submitting code & software for further information.

Data

Policy information about availability of data

All manuscripts must include a data availability statement. This statement should provide the following information, where applicable:

- Accession codes, unique identifiers, or web links for publicly available datasets
- A description of any restrictions on data availability
- For clinical datasets or third party data, please ensure that the statement adheres to our policy

Atomic coordinates and the cryo-EM map have been deposited in the PDB and the EM Data Bank, respectively, under following accession numbers; EMD-31351 and 7EWL (apo GPR158), EMD-31360 (overall refined 2GPR158-RGS7-Gβ5), EMD-31365 (locally refined 2GPR158-RGS7-Gβ5), and 7EWP (2GPR158-RGS7-Gβ5), and EMD-31363 (overall refined 2GPR158-2RGS7-2Gβ5), EMD-31366 (locally refined 2GPR158-2RGS7-2Gβ5), and 7EWR (2GPR158-2RGS7-2Gβ5). The reported structural model of Bos taurus RGS7-Mus musculus Gβ5 (PDB 6N9G) was used as an initial template to build the 2GPR158-RGS7-Gβ5 model.

Field-specific reporting

Please select the one below that is the best fit for your research. If you are not sure, read the appropriate sections before making your selection.

☒ Life sciences ☐ Behavioural & social sciences ☐ Ecological, evolutionary & environmental sciences

For a reference copy of the document with all sections, see nature.com/documents/nr-reporting-summary-flat.pdf

Life sciences study design

All studies must disclose on these points even when the disclosure is negative.

Sample size	10,361 movies of apo GPR158 and 20,613 movies of GPR158-RGS7-Gβ5 complex were acquired for structural determination using a Talos Arctica electron microscope (FEI) equipped with a Gatan K3 summit direct electron detector. All cryo-EM maps generated from the movies for apo GPR158 (at a global resolution of 3.52 Å) and GPR158-RGS7-Gβ5 (the 2:1:1 complex at 4.31 Å and the 2:2:2 at 4.68 Å) were sufficient for atomic model building and structural fitting, respectively. For adenylate cyclase activation cell-based assay, at least three biologically independent experiments were conducted and average of multiple experiments was represented as indicated in related Figure legends.
Data exclusions	No data was systematically excluded. The procedure of generating 3D maps from cryo-EM particles involves sorting for particles that are false-positively picked, damaged or in minority conformations that are unlikely to refine correctly. This is implemented in Relion v. 3.0, CryoSparc v. 2.15 and v. 3.1.
Replication	Adenylate cyclase activation cell-based assays were repeated independently three times. All attempts at replication were successful.<br>The data are presented as mean values with standard deviations from three independent experiments. The differences between the mean values were analyzed one-way ANOVA and Tukey's post-hoc test ($n = 3$; $F5, 12 = 157.2$; $*p < 0.05$, $**p < 0.01$, $***p < 0.001$, $****p < 0.0001$).<br>Western blot analysis were repeated independently twice with similar results. Quantification data are presented as mean values with standard deviations from two independent experiments. All attempts at replication were successful.
Randomization	Randomization was not relevant to our study as there were no groups allocated in cryo-EM and cell-based assay data. Extracted particles for generating 3D maps were randomly assigned to calculate gold-standard FSC.
Blinding	Blinding was not required for this study because no subjective allocation was involved.

Reporting for specific materials, systems and methods

We require information from authors about some types of materials, experimental systems and methods used in many studies. Here, indicate whether each material, system or method listed is relevant to your study. If you are not sure if a list item applies to your research, read the appropriate section before selecting a response.

Materials & experimental systems

n/a	Involved in the study	
☐	☒	Antibodies
☐	☒	Eukaryotic cell lines
☒	☐	Palaeontology and archaeology
☒	☐	Animals and other organisms
☒	☐	Human research participants
☒	☐	Clinical data
☒	☐	Dual use research of concern

Methods

n/a	Involved in the study	
☒	☐	ChIP-seq
☒	☐	Flow cytometry
☒	☐	MRI-based neuroimaging

Antibodies

Antibodies used	anti-OctA antibody (Santa Cruz Biotechnology, 1:400 dilution), anti-GFP antibody (Santa Cruz Biotechnology, 1:40 dilution), anti-glyceraldehyde-3-phosphate dehydrogenase antibody (GAPDH; Sigma, 1:5000 dilution) and anti-N-cadherin antibody (Sigma, 1:50 dilution), anti-mouse IgG secondary antibody (Santa Cruz Biotechnology, 1:2000 dilution)
Validation	OctA (Cat #: sc-166355, mouse): https://www.scbt.com/p/octa-probe-antibody-h-5<br>GFP (Cat #: sc-9996, mouse): https://www.scbt.com/p/gfp-antibody-b-2<br>GAPDH (Cat #: MAB374, mouse): https://www.merckmillipore.com/KR/ko/product/Anti-Glyceraldehyde-3-Phosphate-Dehydrogenase-Antibody-clone-6C5,MM_NF-MAB374?ReferrerURL=https%3A%2F%2Fwww.google.com%2F<br>N-Cadherin (Cat #: C3865, mouse): https://www.sigmaaldrich.com/KR/ko/product/sigma/c3865<br>anti-mouse IgG secondary antibody (Cat #: c-516102, mouse): https://www.scbt.com/ko/p/m-igg-kappa-bp-hrp

Eukaryotic cell lines

Policy information about cell lines

Cell line source(s)	Cell lines were purchased from the American Type Culture Collection (ATCC): HEK293S GnTI-(CRL-3022), HEK293T (CRL-3216) and insect Sf9 (CRL-1711).
Authentication	The cell lines were authenticated by ATCC. Cellular morphology and growth characteristics were checked for each passage of cells.
Mycoplasma contamination	Cell lines were certified by ATCC. The cell lines were not tested for mycoplasma contamination.
Commonly misidentified lines (See ICLAC register)	None.
